# Supplementary material for: Cellulose Nanocrystals Crosslinked with Sulfosuccinic Acid as Sustainable Proton Exchange Membranes for Electrochemical Energy Applications
Source: Membranes (Basel). 2022 Jun 26;12(7):658. doi: 10.3390/membranes12070658 (PMC9319731; doi:10.3390/membranes12070658)
Supplement: Supplementary file 1 [file membranes-12-00658-s001.zip › membranes-1780041-supplementary.pdf]

Supplementary Material

Cellulose nanocrystals crosslinked with sulfosuccinic acid as sustainable proton exchange membranes for electrochemical energy applications

Olena Selyanchyn [1], Thomas Bayer [2], Dino Klotz [3], Roman Selyanchyn [3,4,5,*], Kazunari Sasaki [3,4,6,7] and Stephen Matthew Lyth [1,3,6,7,8,*]

[1] Department of Automotive Science, Graduate School of Integrated Frontier Sciences, Kyushu University, 744 Motooka, Nishi-ku, 819-0395, Fukuoka, Japan; olenaselyanchyn@gmail.com
[2] Lloyd’s Register Group Limited, Queens Tower A10F. 2-3-1, Minatomirai, Nishi-Ku Yokohama, 220-0012 Japan; bayerthomas@gmx.net
[3] International Institute for Carbon-Neutral Energy Research (WPI-I²CNER), Kyushu University, 744 Motooka, Nishi-ku, 819-0395, Fukuoka, Japan; dino.klotz@i2cner.kyushu-u.ac.jp
[4] Kyushu University Platform for Inter/Transdisciplinary Energy Research (Q-PIT), 744 Motooka, Nishi-ku, 819-0395, Fukuoka, Japan; romanselyanchyn@i2cner.kyushu-u.ac.jp
[5] Research Center for Negative-Emissions Technologies (K-NETs), Kyushu University, 744 Motooka, Nishi-ku, 819-0395, Fukuoka, Japan
[6] Next-Generation Fuel Cell Research Center (NEXT-FC), Kyushu University, 744 Motooka, Nishi-ku, 819-0395, Fukuoka, Japan; sasaki.kazunari.278@m.kyushu-u.ac.jp
[7] International Research Center for Hydrogen Energy (HY30), Kyushu University, 744 Motooka, Nishi-ku, 819-0395, Fukuoka, Japan; lyth@i2cner.kyushu-u.ac.jp
[8] Department of Mechanical Engineering, University of Sheffield, Western Bank, Sheffield S1 3JD, United Kingdom
* Correspondence: romanselyanchyn@i2cner.kyushu-u.ac.jp (R.S.); lyth@i2cner.kyushu-u.ac.jp (S.M.L.)

Photos of the membrane samples

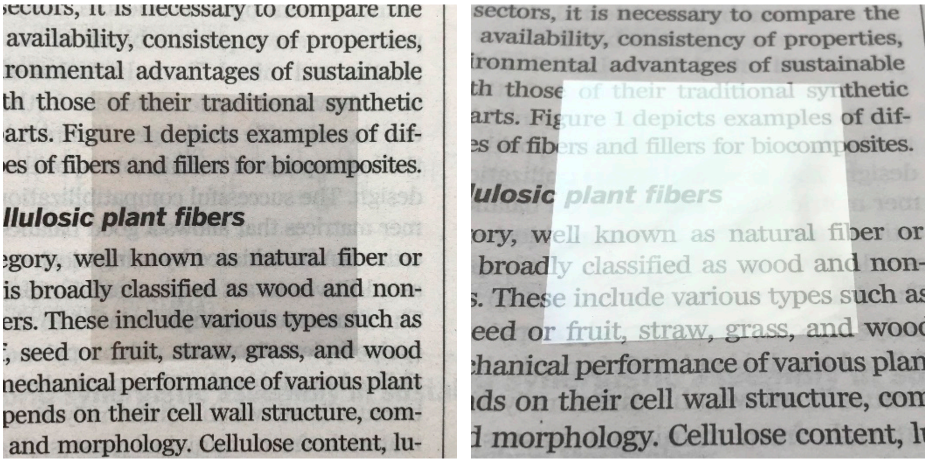

(a) CNC

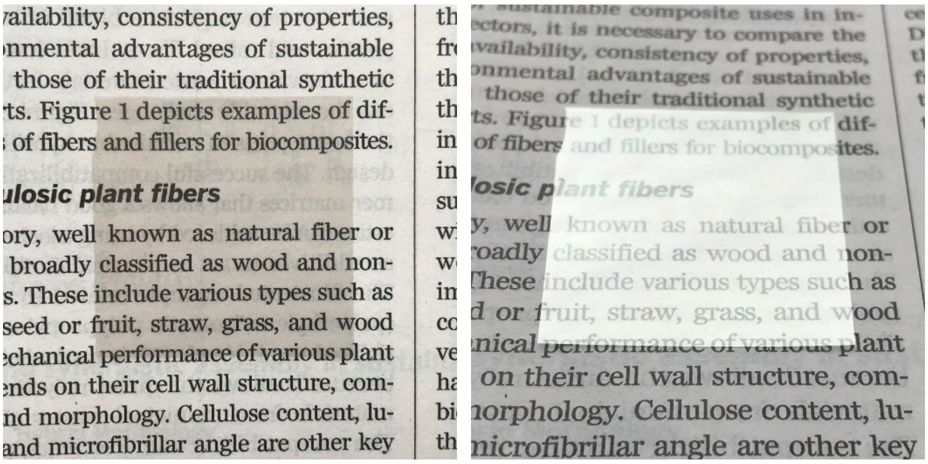

(b) CNC-1%-SSA

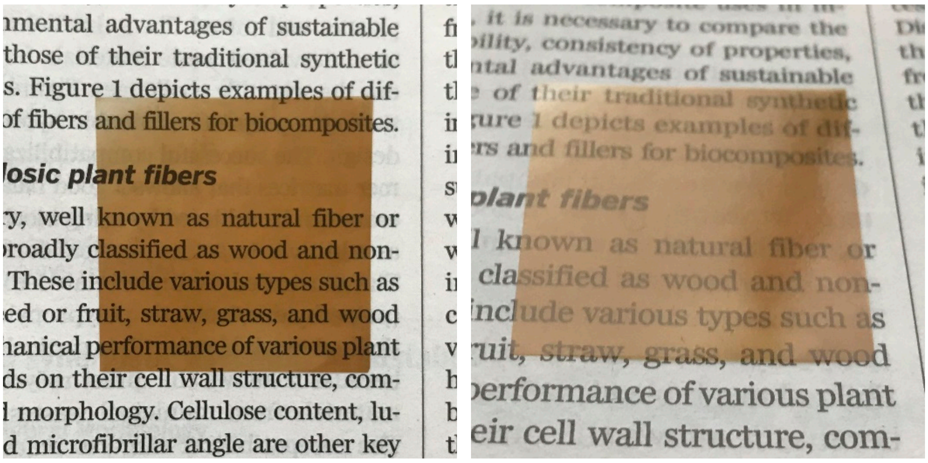

(c) CNC-3%-SSA

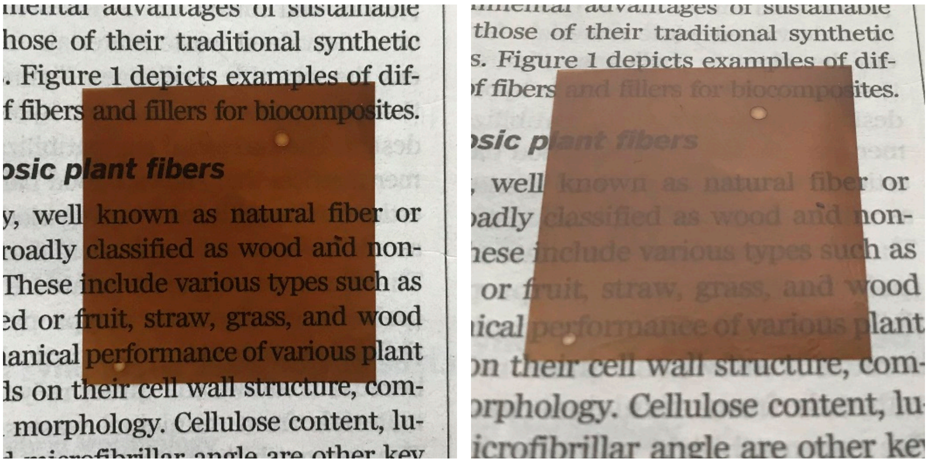

(d) CNC-5%-SSA

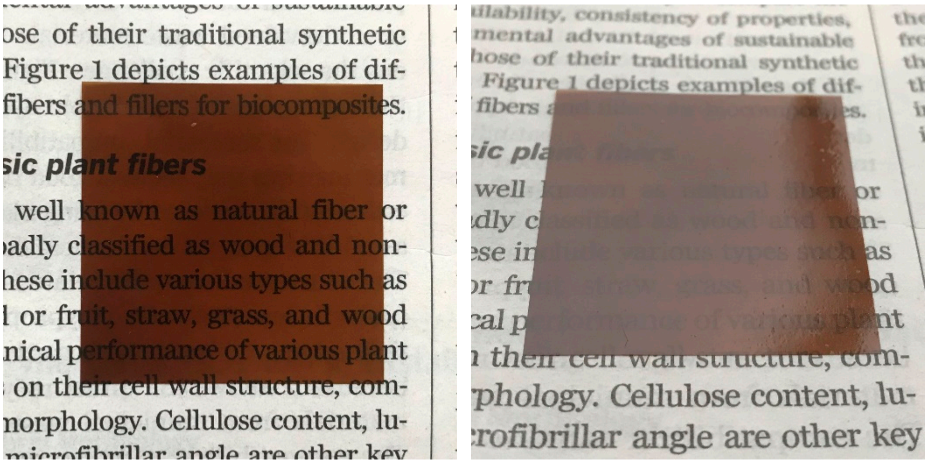

(e) CNC-7%-SSA

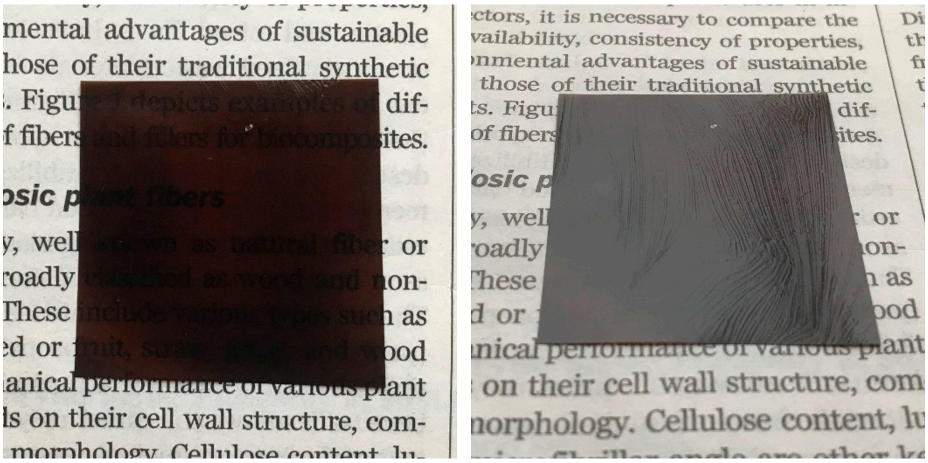

(f) CNC-10%-SSA

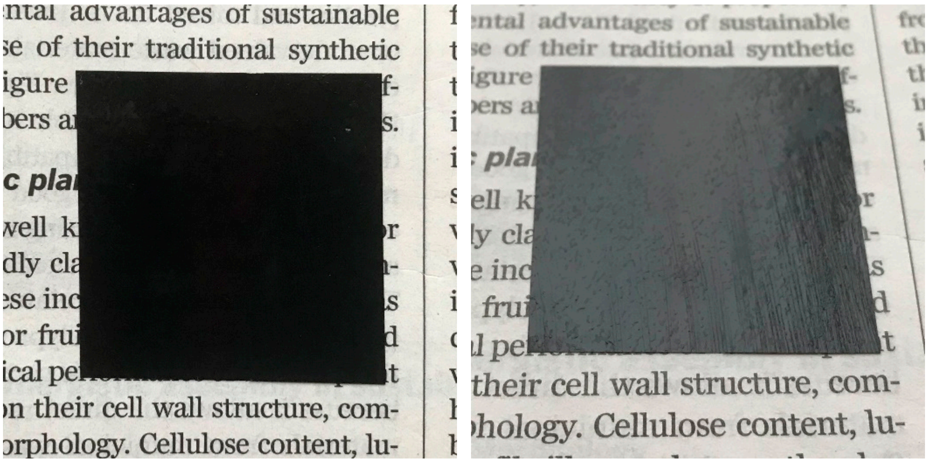

(g) CNC-15%-SSA

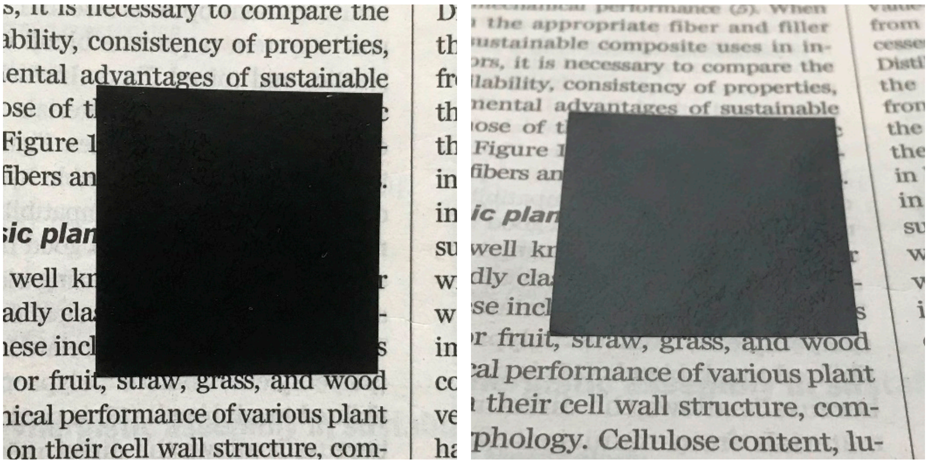

(h) CNC-20%-SSA

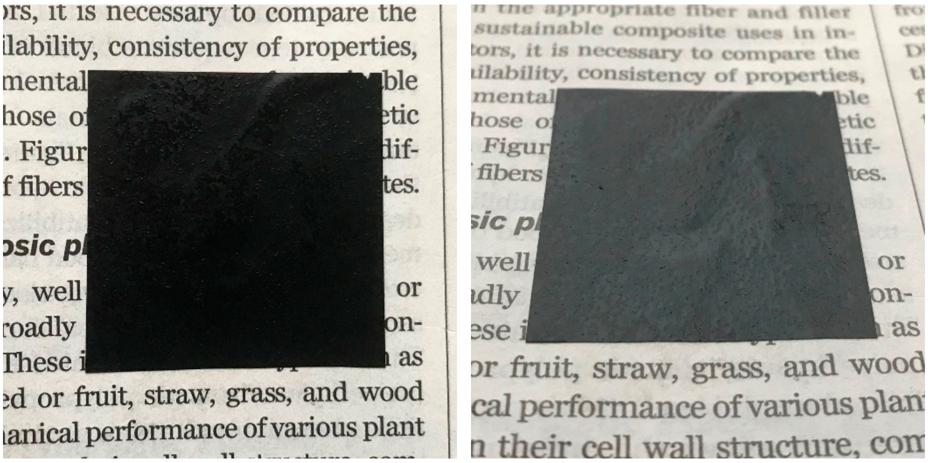

(i) CNC-25%-SSA

Figure S1. Photographs (straight and tilted view) of a pure cellulose nanocrystal membrane (CNC), and eight membranes crosslinked using different proportions of sulfosuccinic acid (SSA). The background text is taken from (Mohanty et al., 2018) to demonstrate the degree of transparency.

Mohanty, A.K.; Vivekanandhan, S.; Pin, J.-M.; Misra, M. Composites from renewable and sustainable resources: Challenges and innovations. *Science* **2018**, *362*, 536–542. https://doi.org/10.1126/science.aat9072.

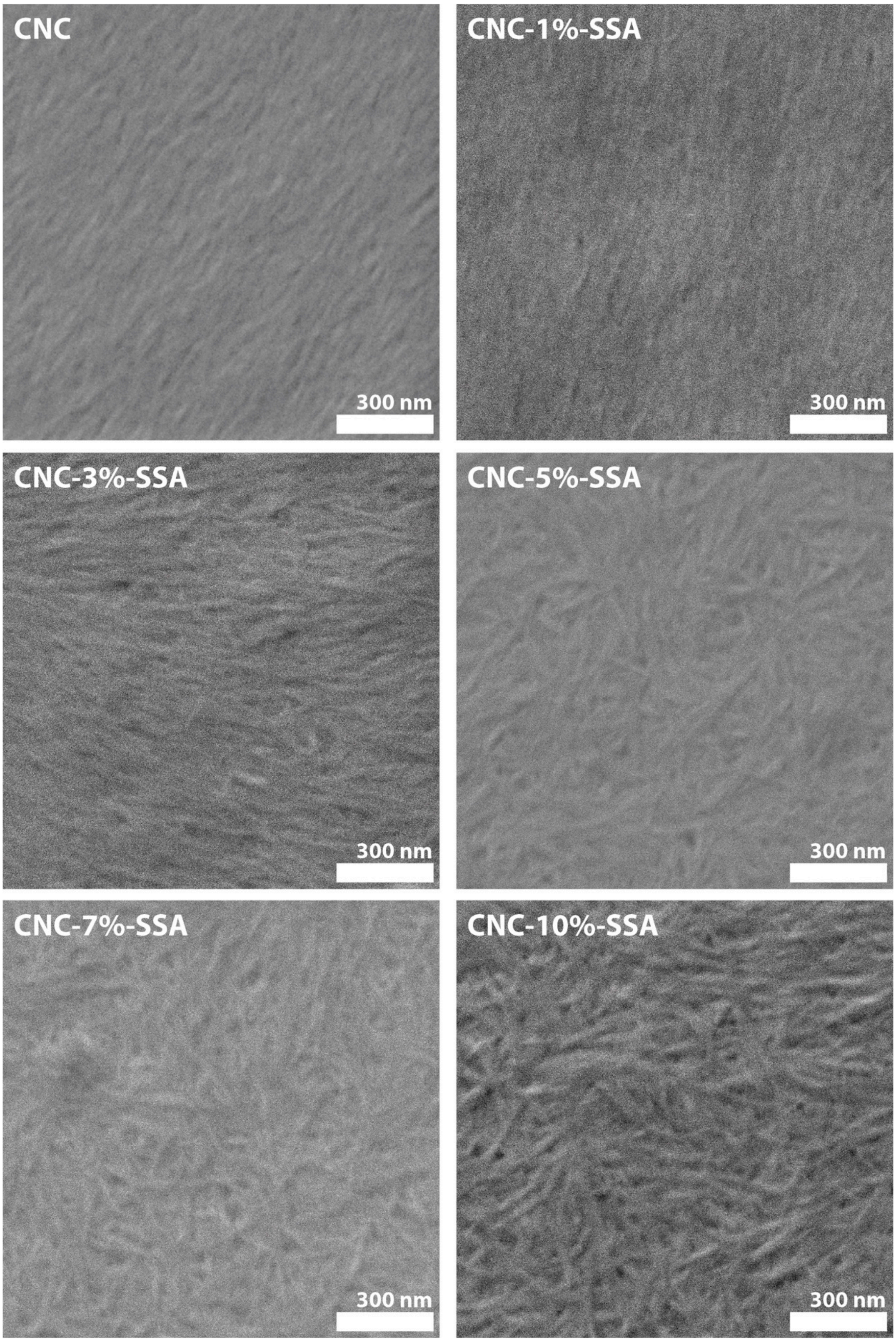

Figure S2. Scanning electron microscopy images of the surface of CNC, and crosslinked CNC-*x*%-SSA membranes demonstrating the concentration of acid at which the crystallites alignment is changed.

Table S1. Peak areas of the main peaks detected in the XRD pattern for the different samples.

	Peak area (a.u)	
	(101)	(002)
CNC	2.04	2.73
CNC-10%-SSA	1.74	2.66
CNC-15%-SSA	1.56	2.70
CNC-20%-SSA	1.79	2.64
CNC-25%-SSA	1.43	2.73
CNC-30%-SSA	1.68	2.64

* Before the calculation of the peak areas XRD patterns for all samples were normalized. Area under curves in the region of 10-30° (2θ) was calculated using the OriginLab data analysis software.

Table S2. Atomic concentration of elements in membranes based on XPS

	**S, at%**	**C, at%**	**O, at%**	**Ratio C/O**
CNC	0.63	71.35	28.02	2.55
CNC-10%-SSA	0.66	70.03	29.31	2.39
CNC-15%-SSA	1.14	68.01	30.85	2.20
CNC-20%-SSA	1.06	69.59	29.35	2.37
CNC-25%-SSA	1.06	68.89	30.05	2.29
CNC-30%-SSA	0.73	69.80	29.47	2.37
CNC-35%-SSA	0.88	70.32	28.80	2.44
CNC-40%-SSA	1.16	71.05	27.79	2.56

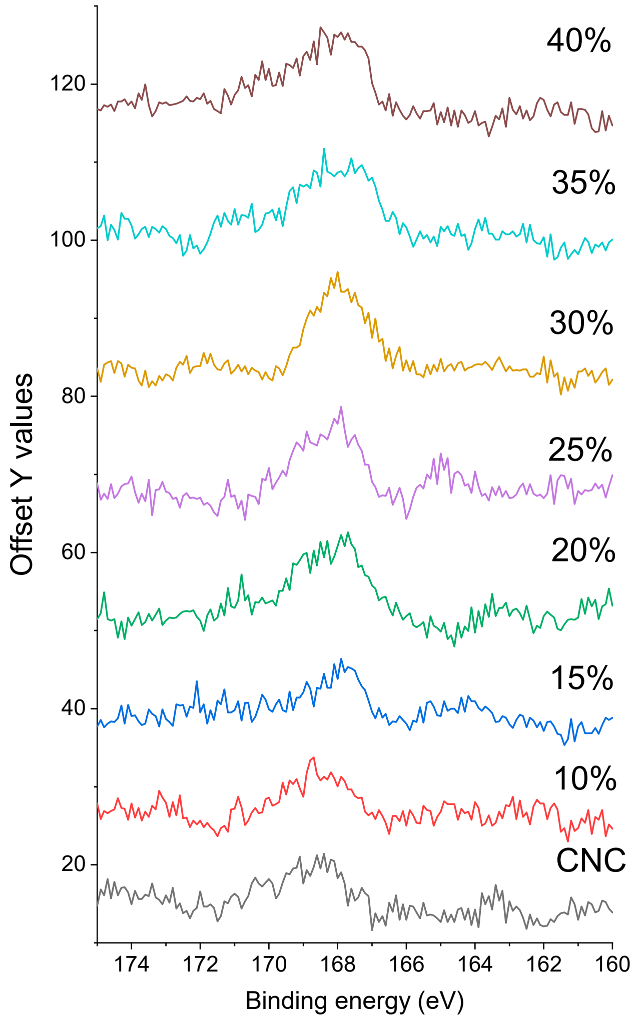

Figure S3. Narrow scan XPS for S 2p signal.

Stress-vs. strain for all samples

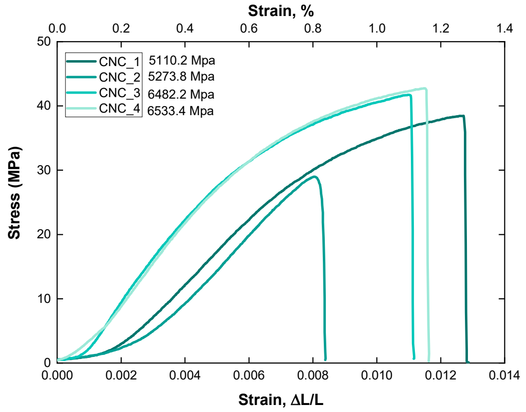

(a) CNC

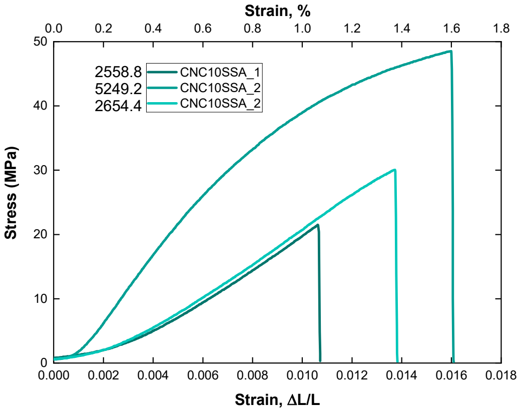

(b) CNC-10%-SSA

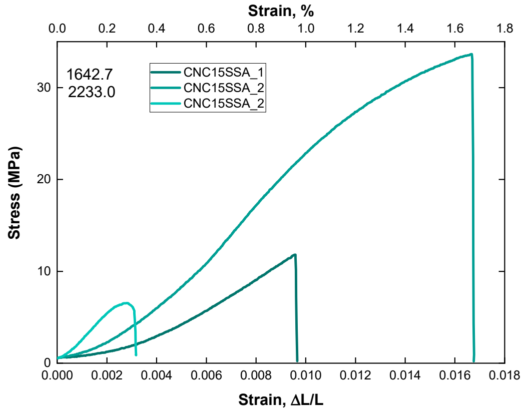

(c) CNC-15%-SSA

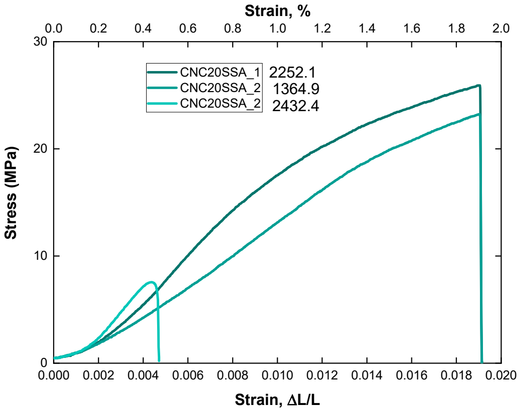

(d) CNC-20%-SSA

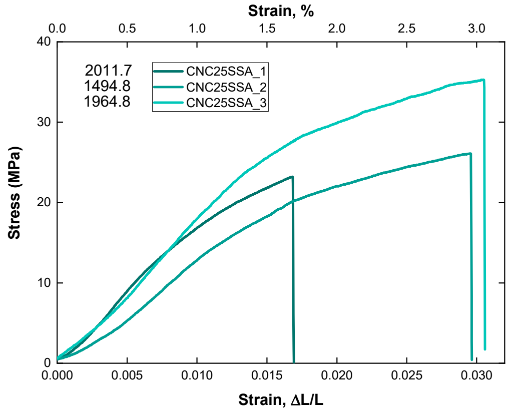

(e) CNC-25%-SSA

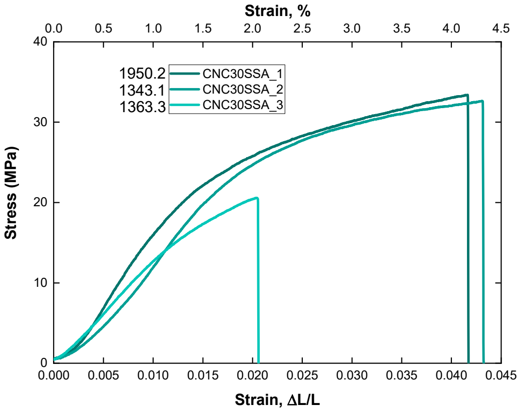

(f) CNC-30%-SSA

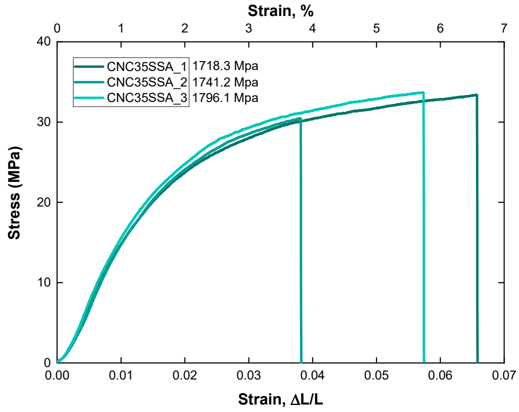

(g) CNC-35%-SSA

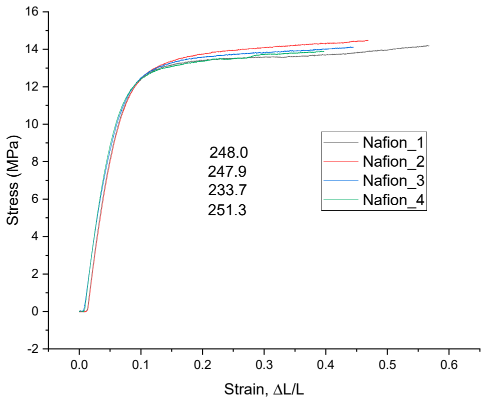

(h) Nafion-212

Figure S4. All data for conducted mechanical tests for (a) pure cellulose nanocrystal membrane (CNC), (b-g) six membranes crosslinked using different proportions of sulfosuccinic acid (SSA) and (h) Nafion-212 membrane measured in the same conditions.

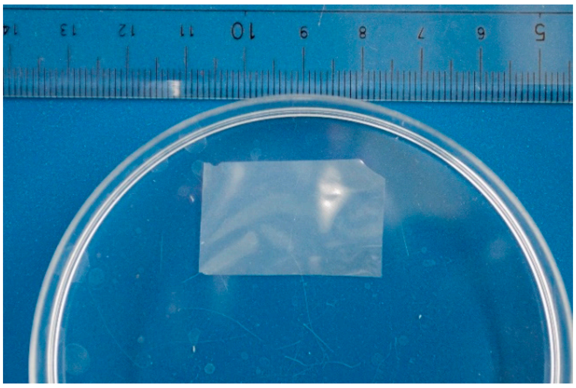

(a)

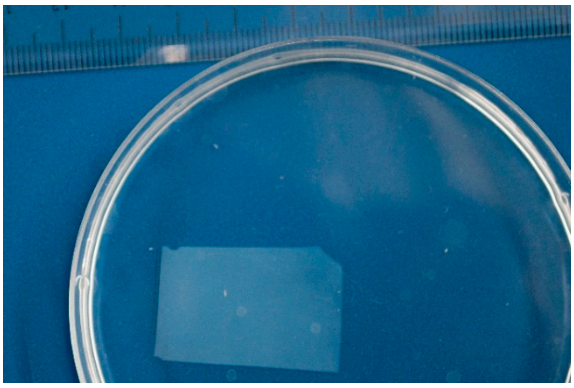

(b)

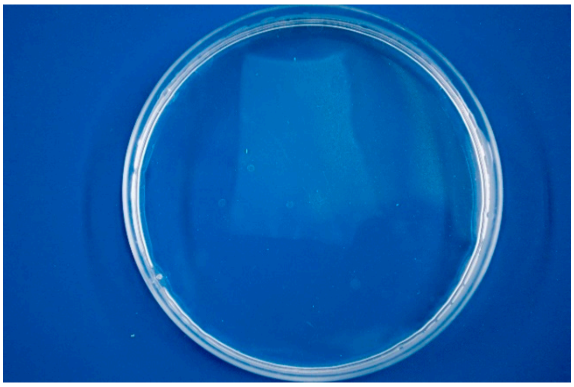

(d)

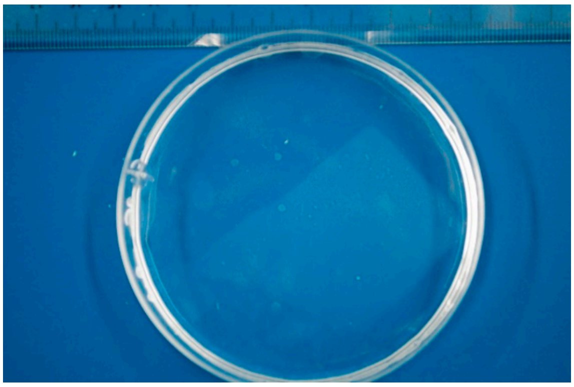

(d)

Figure S5. Photo of the CNC membrane (a) dry; (b) immediately after immersion in water; (c) 10 min after immersion in water; (d) 20 min after immersion in water.

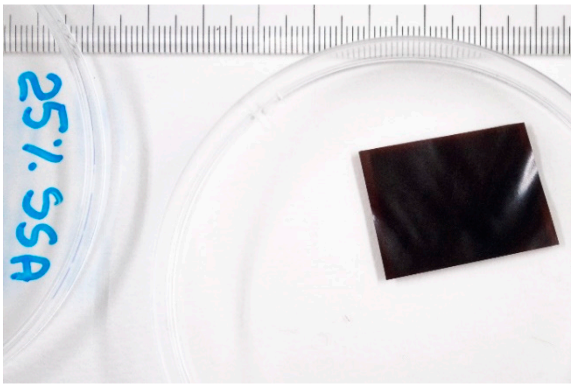

(a)

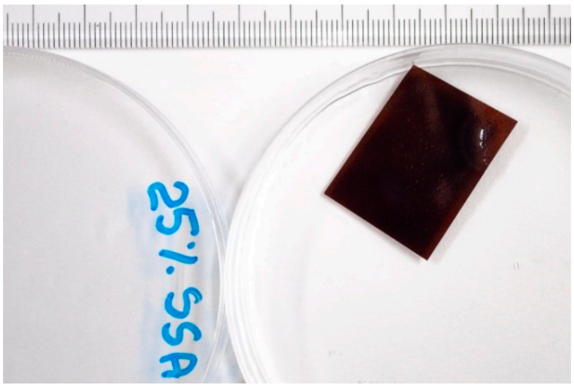

(b)

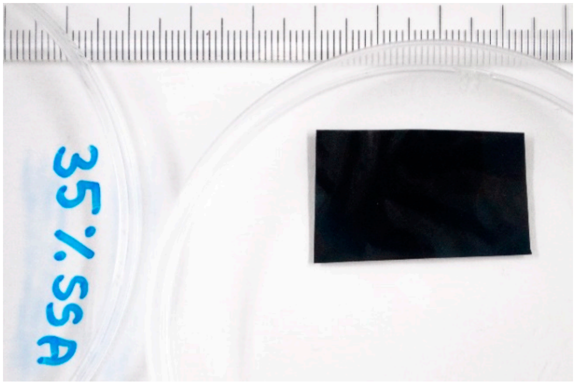

(c)

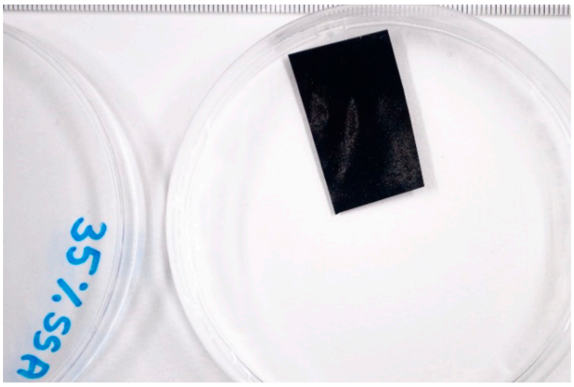

(d)

Figure S6. Photo of the CNC-25%-SSA membrane (a) dry; (b) ~20 min after immersion in water and CNC-355%-SSA membrane (c) dry; (d) ~20 min after immersion in water.

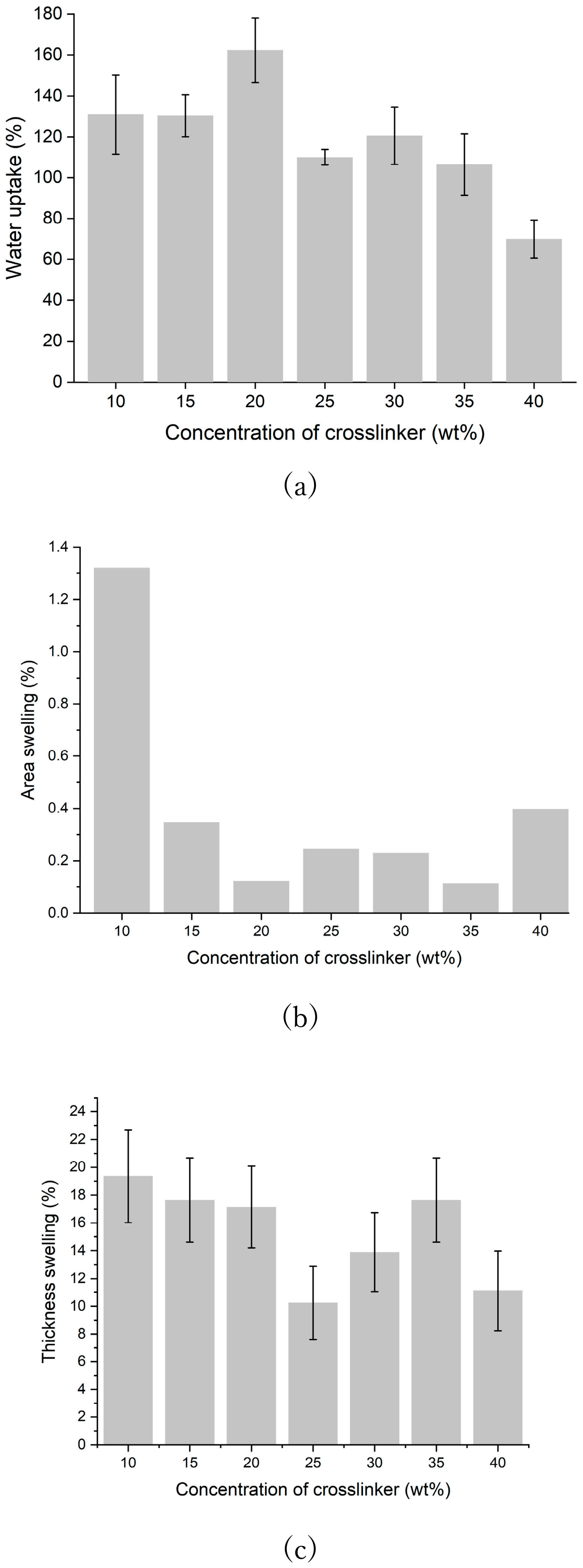

Figure S7. Characterization of the membranes swelling in water (a) mass uptake; (b) in-plane (area) swelling; (c) through-plane (thickness) swelling.

Titration curves for all samples

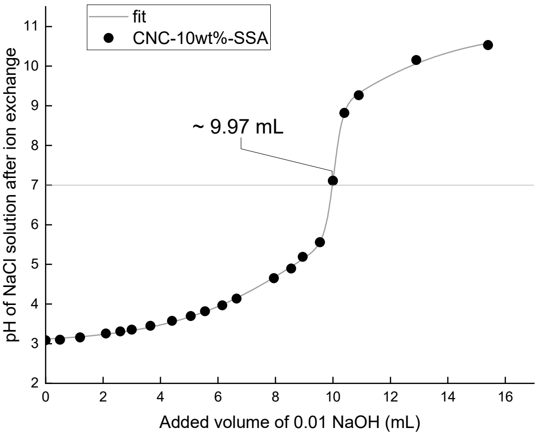

(a)

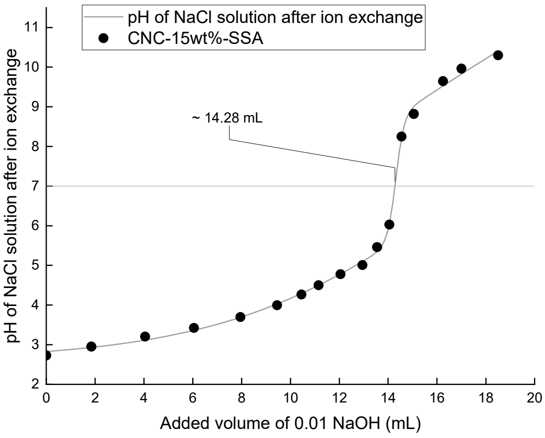

(b)

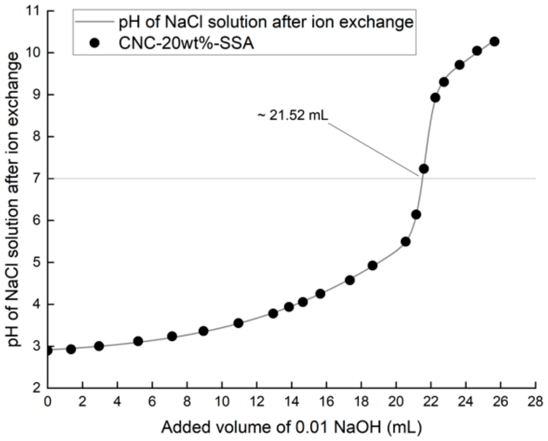

(c)

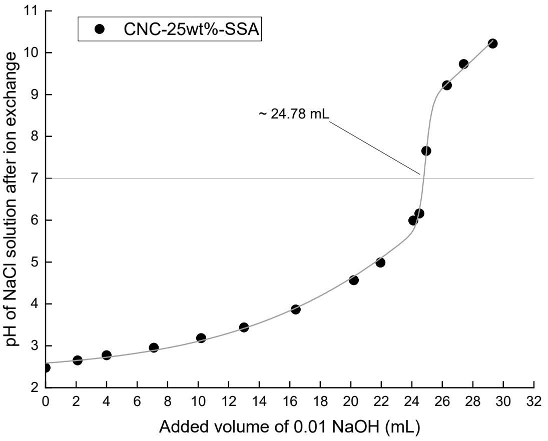

(d)

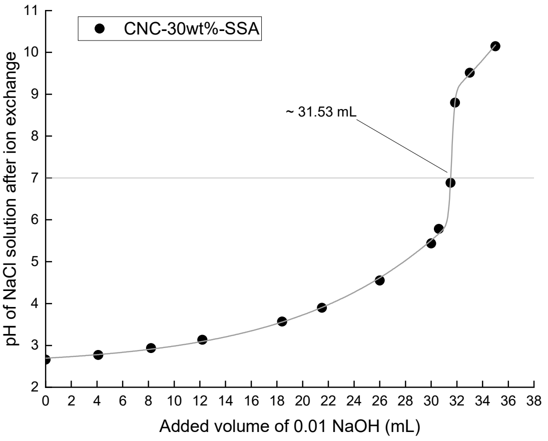

(e)

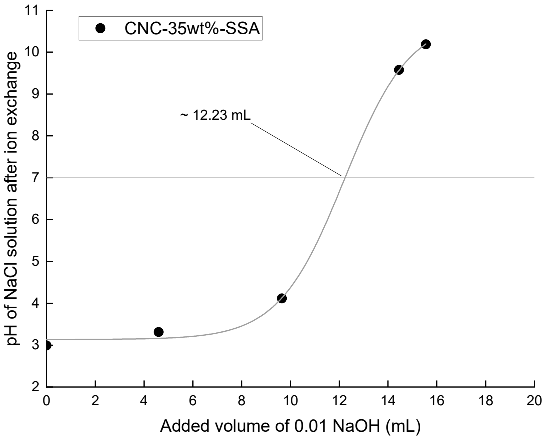

(f)

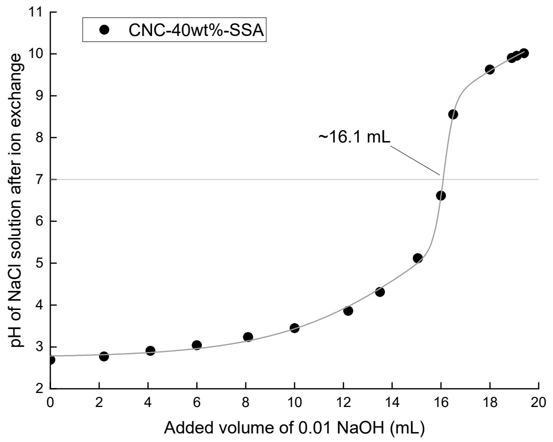

(g)

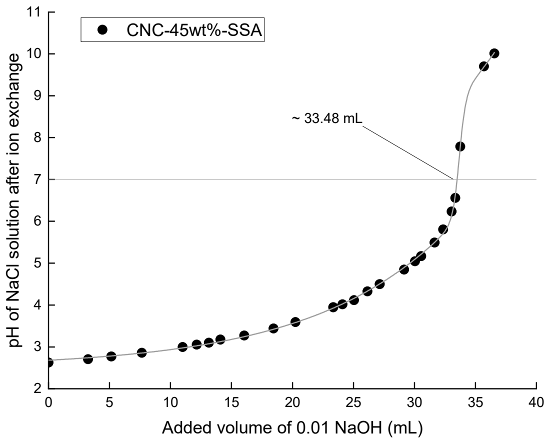

(h)

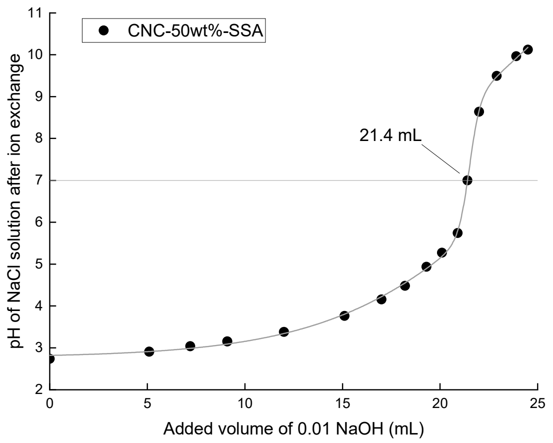

(i)

Figure S8. (a-i) Titration curves obtained for CNC-x%-SSA membranes (x = 10 to 50%) incubated in 1 M NaCl solution. The equivalent volume of the NaOH solution was estimated from the titration curve intercept with a reference line of pH = 7.

Impedance Spectra

Representative through-plane impedance spectra of pristine CNC and crosslinked CNC-25%-SSA membranes are shown in Fig. S9, measured at different RH. At low RH, the impedance spectra are represented by semicircle at high frequency, with a maximum amplitude corresponding the ohmic resistance of the membrane (Fig. S9a and S9d). For CNC, this semicircle remains as the RH increases. Regarding the dependance on humidity, at 40 °C the ohmic resistance of CNC decreases by three orders of magnitude as the RH increases, and this regular decrease is generally associated with protonic conduction (Gadim et al. 2017). The influence of temperature is less dramatic - at 95% RH only a four-fold decrease in resistance is measured between 40 and 120°C for the CNC membrane. Similar tendencies are observed for CNC-25%-SSA (Fig. S9 d-f). In this case, the high-frequency semicircle disappears as the RH increases, so in this case the ohmic resistance corresponds to the extrapolated high-frequency intercept with the real axis. In the crosslinked membrane, the measured resistances are consistently almost two-orders of magnitude lower than in pristine CNC. This is attributed to the higher proportion of acidic sulfonic groups. At 40 ºC, the ohmic resistance decreases by three orders of magnitude as the RH increases from 30% to ~95%RH. Meanwhile, in the case of CNC-25%-SSA the influence of the temperature is more pronounced at lower RH and less pronounced at high RH, varying between ~2.0 Ω at 40 ºC, 0.8 Ω at 80 ºC, and 0.7 Ω at 120 °C.

Gadim, T. D. O.; Loureiro, F. J. A.; Vilela, C.; Rosero-Navarro, N.; Silvestre, A. J. D.; Freire, C. S. R.; Figueiredo, F. M. L. Protonic Conductivity and Fuel Cell Tests of Nanocomposite Membranes Based on Bacterial Cellulose. *Electrochim. Acta* **2017**, *233*, 52–61. https://doi.org/10.1016/j.electacta.2017.02.145.

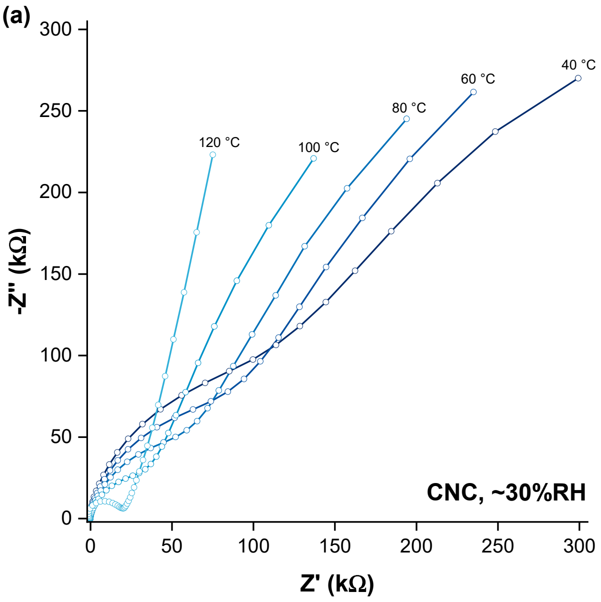
(a)
CNC, ~30%RH
120 °C
100 °C
80 °C
60 °C
40 °C
-Z'' (kΩ)
Z' (kΩ)

(a)

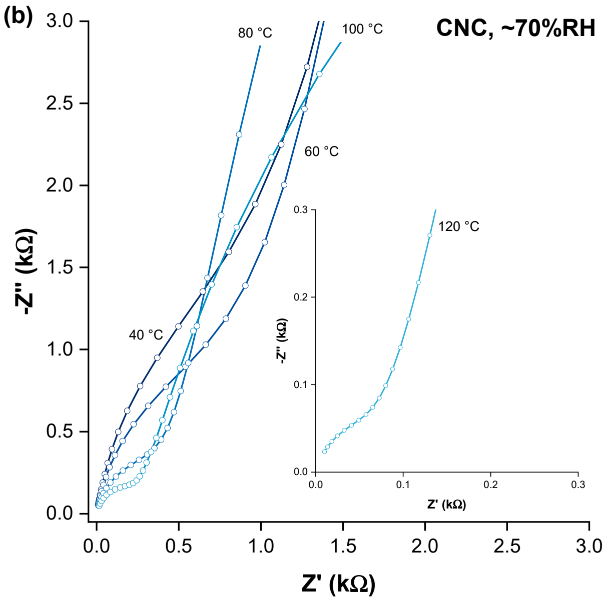
(b)
CNC, ~70%RH
80 °C
100 °C
60 °C
40 °C
120 °C
-Z'' (kΩ)
Z' (kΩ)

(b)

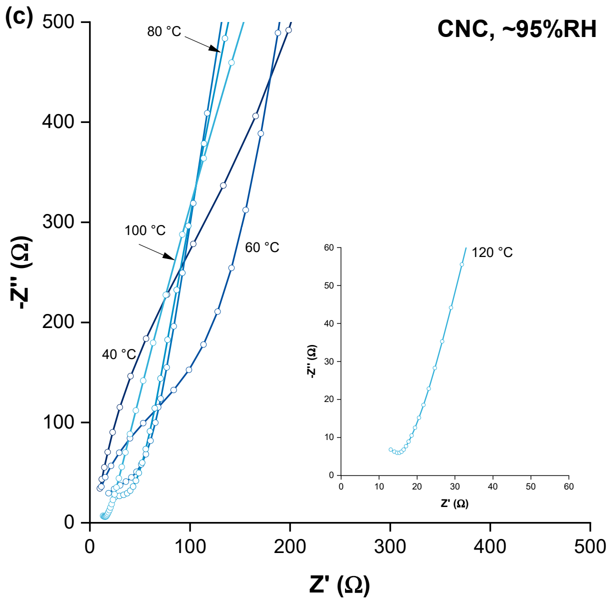
(c)
CNC, ~95%RH
80 °C
100 °C
60 °C
40 °C
120 °C
-Z'' (Ω)
Z' (Ω)

(c)

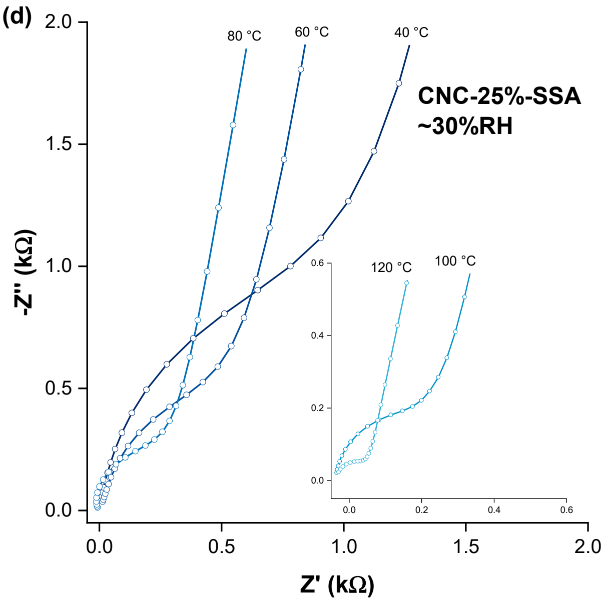
(d)
CNC-25%-SSA
~30%RH
80 °C
60 °C
40 °C
120 °C
100 °C
-Z'' (kΩ)
Z' (kΩ)

(d)

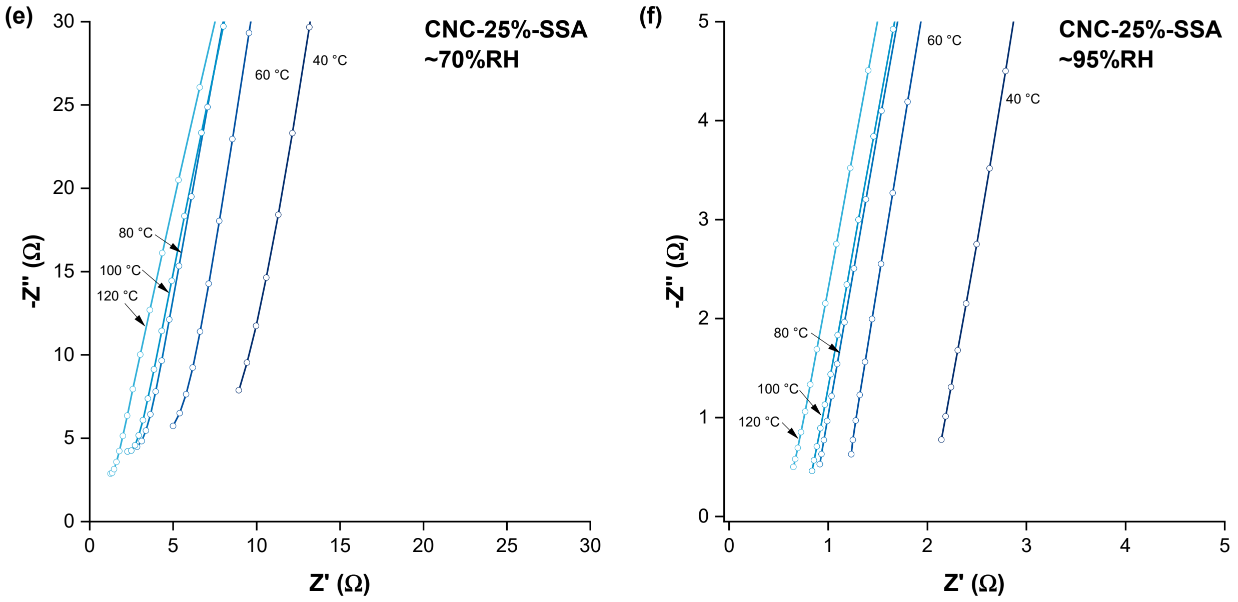

(e) (f)

Figure S9. Nyquist plots of the CNC (a, b, c) and CNC-25%-SSA (d, e, f) membranes collected at different temperatures (40-120°C) in the through-plane configuration and at relative humidity of *ca.*30%, *ca.*70% and *ca.*95% respectively (as indicated on the plots). Thickness of both membranes was ~ 35 microns. Please note the different scales used in each case.

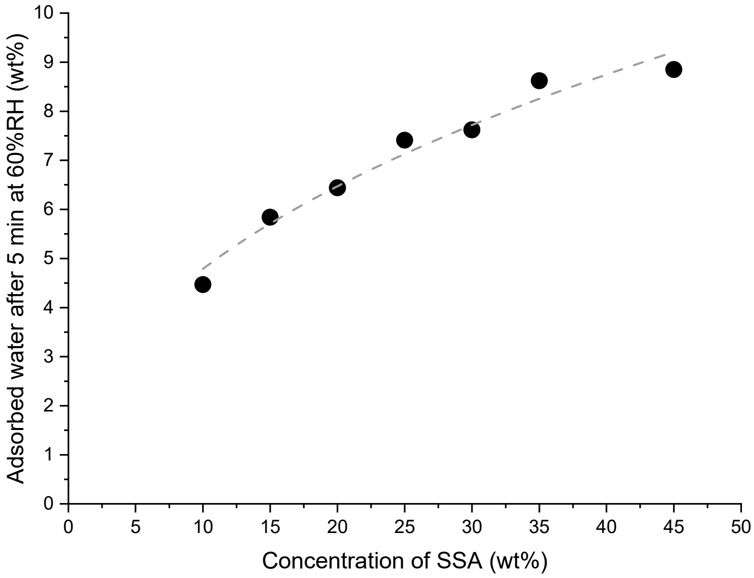

Figure S10. Sorption of water in CNC-x%-SSA membranes extracted from comparison of membrane mass immediately after vacuum drying and 5-min incubation in ambient environment (~60% RH, 25 °C)
